# Supplementary material for: Forelimb muscle and joint actions in Archosauria: insights from Crocodylus johnstoni (Pseudosuchia) and Mussaurus patagonicus (Sauropodomorpha)
Source: PeerJ. 2017 Nov 24;5:e3976. doi: 10.7717/peerj.3976 (PMC5703147; doi:10.7717/peerj.3976)
Supplement: Supplemental Information 1 [file peerj-05-3976-s001.docx]

**Table S1**. Wrapping surfaces used for the musculoskeletal models of *Crocodylus johnstoni* and *Mussaurus patagonicus*.

| *Crocodylus* |  |  |  |  |  |  |  |  |  |  |
| --- | --- | --- | --- | --- | --- | --- | --- | --- | --- | --- |
| Muscle involved | Location | Shape | r(x) | r(y) | r(z) | t(x) | t(y) | t(z) | Radius | Height |
| DS | Glenoid/scapula | Cylinder | 19.73 | -54.22 | -30.11 | 0.0666 | -0.0949 | -0.1011 | 0.0100 | 0.0804 |
| DC, CBD | Proximal humerus | Cylinder | 39.17 | -7.85 | 35.64 | 0.0257 | 0.0081 | -0.0013 | 0.0140 | 0.0600 |
| CBV | Proximal humerus | Cylinder | -49.32 | 65.54 | 60.72 | 0.0012 | 0.0074 | -0.0032 | 0.0170 | 0.1000 |
| TBM2, TBL | Distal humerus | Cylinder | 20.42 | 63.19 | 169.94 | -0.0317 | -0.1920 | 0.0198 | 0.0180 | 0.1000 |
| TBC, TBS | Distal humerus | Cylinder | 20.42 | 63.19 | 127.35 | -0.066 | -0.1923 | 0.0112 | 0.0420 | 0.0800 |
| TBM1, TBM3, TBM4 | Distal humerus | Cylinder | 21.21 | 62.58 | -57.29 | -0.0316 | -0.1913 | 0.0190 | 0.0200 | 0.0600 |
| BB | Proximal humerus | Torus | 91.47 | -0.79 | 115.70 | 0.0155 | 0.0062 | -0.0610 | 0.0160/0.0560 |  |
| ECR | Distal humerus | Cylinder | 179.86 | -41.57 | -176.87 | -0.0032 | -0.1930 | 0.0189 | 0.0180 | 0.0800 |
| SU | Distal humerus | Cylinder | 36.08 | 28.95 | 147.80 | 0.0357 | -0.0444 | 0.0072 | 0.0120 | 0.0600 |
| *Mussaurus* |  |  |  |  |  |  |  |  |  |  |
| Muscle involved | Location | Shape | r(x) | r(y) | r(z) | t(x) | t(y) | t(z) | Radius | Height |
| BB | Humeral shaft | Ellipsoid | -22.50 | 10.55 | -91.08 | 0.1962 | 0.0284 | 0.0026 | 0.0156/0.039/0.0585 |  |
| BB, SUC | Proximal scapulocoracoid | Ellipsoid | 74.18 | -3.99 | -40.55 | 0.90 | -0.14 | 0.070 | 0.039/0.009/0.129 |  |
| DC | Thorax | Ellipsoid | -117.67 | 19.22 | -8.24 | 0.0676 | -0.3229 | 0.0092 | 0.0312/0.195/0.117 |  |
| DS | Thorax | Ellipsoid | -123.59 | -70.08 | -107.42 | 0.0484 | -0.3974 | 0.0019 | 0.117/0.156/0.039 |  |
| SHP | Thorax | Ellipsoid | 179.62 | -11.71 | 8.19 | 0.0831 | -0.2634 | -0.0332 | 0.0195/0.078/0.078 |  |
| TBC, TBM1 | Distal humerus | Cylinder | -13.22 | 3.87 | 73.46 | 0.4005 | -0.0056 | -0.0042 | 0.0312 | 0.195 |
| TBM2 | Distal humerus | Cylinder | 165.23 | -0.54 | 70.06 | 0.3991 | -0.0058 | -0.0090 | 0.0312 | 0.195 |
| TBL, TBM4 | Distal humerus | Cylinder | 168.57 | -2.62 | -109.10 | 0.3998 | -0.0034 | -0.0096 | 0.0312 | 0.0975 |
| TBS | Distal humerus | Cylinder | -12.14 | 2.80 | -88.58 | 0.3990 | -0.0060 | -0.0087 | 0.0312 | 0.195 |
| TBM3 | Distal humerus | Cylinder | -9.07 | 1.88 | -86.43 | 0.4009 | -0.0014 | 0.0044 | 0.0312 | 0.1957 |
| TM | Thorax | Ellipsoid | -50.06 | -67.95 | -36.96 | 0.0445 | -0.3835 | -0.0371 | 0.156/0.156/0.039 |  |

‘Shape’ identifies the wrapping surface’s geometry. Rotations around the x, y and z axes (from the segment’s origin) are r(x), r(y) and r(z), and translations (of the surface’s centroid relative to the segment’s origin) are t(x), t(y) and t(z). The ‘‘Radius’’ and ‘‘Height’’ columns give object dimensions (in metres). For additional muscle abbreviations see Table 1.
