# Supplementary material for: Forelimb muscle and joint actions in Archosauria: insights from Crocodylus johnstoni (Pseudosuchia) and Mussaurus patagonicus (Sauropodomorpha)
Source: PeerJ. 2017 Nov 24;5:e3976. doi: 10.7717/peerj.3976 (PMC5703147; doi:10.7717/peerj.3976)
Supplement: Supplemental Information 2 — ‘MC II’ is metacarpal II length. Lengths are in metres (m); for Mussaurus the sum circumference in mm was used to estimate body mass (see Discussion), and the ratio of ‘linearized’ body masses to the 0.33exponent is shown in the final column’s final entry (for comparison to normalizing ratios of segment lengths in the ‘ratio’ row to the left). [file peerj-05-3976-s002.docx]

**Table S2**. Assessment of normalizing metrics used in moment arm analyses. ‘MC II’ is metacarpal II length. Lengths are in metres (m); for *Mussaurus* the sum circumference in mm was used to estimate body mass (see Discussion), and the ratio of ‘linearized’ body masses to the ^0.33^ exponent is shown in the final column’s final entry (for comparison to normalizing ratios of segment lengths in the ‘ratio’ row to the left).

| Muscle | Humerus | Radius-ulna | MC II | Humeral circumference in m | Femoral circumference in m | Sum in mm | Body mass in kg | Ratio^0.33^ |
| --- | --- | --- | --- | --- | --- | --- | --- | --- |
| *Crocodylus johnstoni* | 0.182 | 0.143 | 0.049 | – | – |  | 20.19 | 2.70 |
| *Mussaurus patagonicus* | 0.366 | 0.271 | 0.091 | 0.163 | 0.273 | 436 | 1486.43 | 11.14 |
| Ratio | 2.01 | 1.90 | 1.86 | – | – |  | 73.622 | 4.13 |
