# Supplementary material for: Forelimb muscle and joint actions in Archosauria: insights from Crocodylus johnstoni (Pseudosuchia) and Mussaurus patagonicus (Sauropodomorpha)
Source: PeerJ. 2017 Nov 24;5:e3976. doi: 10.7717/peerj.3976 (PMC5703147; doi:10.7717/peerj.3976)
Supplement: Supplemental Information 3 [file peerj-05-3976-s003.docx]

**Table S3**. Ranges of motion (ROMs) of forelimb in *Mussaurus* and *Crocodylus* about each degree of freedom analyzed in this study in the reference pose.

|  | Joint | Pronation (°) | Supination (°) | **Total long-axis rotation (°)** | Abduction (°) | Adduction (°) | **Total ab/ adduction (°)** | Flexion (°) | Extension (°) | **Total flexion/ extension (°)** |
| --- | --- | --- | --- | --- | --- | --- | --- | --- | --- | --- |
| *Mussaurus patagonicus* | Glenohumeral | -25 | 25 | **50** | -25 | 10 | **35** | 0 | -80 | **80** |
|  | Elbow | -30 | 5 | **35** | -5 | 5 | **10** | 130 | 0 | **130** |
|  | Wrist | - | - | **-** | -10 | 10 | **20** | 70 | -30 | **100** |
|  | Metacarpo-phalangeal | - | - | **-** | - | - | **-** | 50 | -40 | **90** |
|  | Interphalangeal | - | - | **-** | - | - | **-** | 70 | -25 | **95** |
| *Crocodylus johnstoni* | Glenohumeral | -20 | 20 | **40** | -5 | 45 | **50** | 5 | -60 | **65** |
|  | Elbow | -20 | 8 | **28** | -5 | 5 | **10** | 110 | 0 | **110** |
|  | Wrist | -10 | 30 | **40** | -30 | 5 | **35** | 40 | -60 | **100** |
