# Supplementary material for: Forelimb muscle and joint actions in Archosauria: insights from Crocodylus johnstoni (Pseudosuchia) and Mussaurus patagonicus (Sauropodomorpha)
Source: PeerJ. 2017 Nov 24;5:e3976. doi: 10.7717/peerj.3976 (PMC5703147; doi:10.7717/peerj.3976)
Supplement: Supplemental Information 4 [file peerj-05-3976-s004.docx]

**Table S4**. Results for glenohumeral joint moment arms (in metres) of major muscle groups in the resting pose for *Mussaurus* and *Crocodylus.*

| Moment arms (m) | | | | | | | | | |
| --- | --- | --- | --- | --- | --- | --- | --- | --- | --- |
|  | Pronation (-)/supination (+) | | | Abduction (-)/adduction (+) | | | Extension (-)/flexion (+) | | |
| *Mussaurus* |  | | |  | | |  | | |
| Muscle | Min | Max | Mean | Min | Max | Mean | Min | Max | Mean |
| DC | 0.0389 | 0.0527 | 0.0483 | -0.0734 | -0.0250 | -0.0525 | 0.0131 | 0.0193 | 0.0166 |
| DS | 0.0431 | 0.0674 | 0.0578 | -0.0155 | -0.0023 | -0.0098 | 0.0045 | 0.0087 | 0.0068 |
| TM | 0.0282 | 0.0559 | 0.0443 | -0.0559 | -0.0307 | -0.0477 | -0.0182 | -0.0078 | -0.0133 |
| SHP | -0.0023 | -0.0018 | -0.0021 | -0.0310 | -0.0161 | -0.0258 | -0.0039 | -0.0004 | -0.0022 |
| SBS | -0.0613 | -0.0510 | -0.0587 | 0.0127 | 0.0179 | 0.0166 | -0.0114 | -0.0003 | -0.0060 |
| SC (all) | 0.0039 | 0.0373 | 0.0163 | 0.0225 | 0.0461 | 0.0392 | 0.0371 | 0.0638 | 0.0528 |
| CBD | 0.0510 | 0.0623 | 0.0592 | -0.0065 | 0.0182 | 0.0063 | 0.0350 | 0.0403 | 0.0389 |
| CBV | -0.0766 | -0.0121 | -0.0551 | 0.0332 | 0.0430 | 0.0405 | 0.0632 | 0.0691 | 0.0674 |
| TBS | -0.0104 | 0.0065 | -0.0021 | -0.1662 | -0.0817 | -0.1278 | -0.0043 | 0.0150 | 0.0054 |
| TBC | -0.0024 | 0.0087 | 0.0034 | -0.0670 | -0.0029 | -0.0360 | -0.0244 | -0.0031 | -0.013 |
| BB | -0.0238 | 0.0340 | -0.0012 | -0.0007 | 0.0469 | 0.0280 | 0.0320 | 0.0700 | 0.0570 |
| *Crocodylus* |  |  |  |  |  |  |  |  |  |
| DC | 0.0212 | 0.0234 | 0.0228 | -0.0343 | -0.0179 | -0.0270 | 0.0229 | 0.0305 | 0.0285 |
| DS | 0.0242 | 0.0270 | 0.0262 | -0.0326 | -0.0257 | -0.0303 | 0.0052 | 0.0207 | 0.0137 |
| TM | 0.0136 | 0.0298 | 0.0232 | -0.0309 | -0.0166 | -0.0261 | -0.0147 | -0.0114 | -0.0138 |
| SHP | 0.0061 | 0.0217 | 0.0150 | -0.0255 | -0.0117 | -0.0207 | -0.0125 | -0.0102 | -0.0118 |
| SBS | -0.0239 | -0.0004 | -0.0134 | -0.0075 | 0.0215 | 0.0081 | -0.0333 | -0.0186 | -0.0298 |
| SC (all) | -0.0001 | 0.0092 | 0.0047 | 0.0078 | 0.0198 | 0.0145 | 0.0245 | 0.0560 | 0.0417 |
| CBD | -0.0008 | 0.0007 | -0.0001 | 0.0020 | 0.0130 | 0.0076 | 0.0172 | 0.0221 | 0.0207 |
| CBV | -0.0177 | -0.0126 | -0.0159 | 0.0248 | 0.0340 | 0.0298 | -0.0143 | 0.0037 | -0.0056 |
| TBS | -0.0029 | 0.0093 | 0.0033 | -0.0400 | -0.0286 | -0.0361 | -0.0373 | -0.0029 | -0.0213 |
| TBC | -0.0035 | 0.0400 | 0.0217 | -0.0347 | -0.0214 | -0.0314 | -0.0514 | -0.0323 | -0.0475 |
| BB | 0.0064 | 0.0116 | 0.0094 | 0.0175 | 0.0209 | 0.0200 | -0.0012 | 0.0309 | 0.0153 |
