# Supplementary material for: Forelimb muscle and joint actions in Archosauria: insights from Crocodylus johnstoni (Pseudosuchia) and Mussaurus patagonicus (Sauropodomorpha)
Source: PeerJ. 2017 Nov 24;5:e3976. doi: 10.7717/peerj.3976 (PMC5703147; doi:10.7717/peerj.3976)
Supplement: Supplemental Information 5 [file peerj-05-3976-s005.docx]

**Table S5**. Results for elbow joint moment arms (in metres) of major muscle groups in the resting pose for *Mussaurus* and *Crocodylus.*

| Moment arms (m) | | | |
| --- | --- | --- | --- |
| Elbow | | | |
| Extension (-)/flexion (+) | | | |
| Muscle | Min | Max | Mean |
| *Mussaurus* |  |  |  |
| Triceps (all) | -0.0642 | -0.0301 | -0.0547 |
| ECR | -0.0300 | 0.0219 | -0.0068 |
| PT | 0.0008 | 0.0074 | 0.0056 |
| AR | -0.0349 | 0.0261 | -0.0131 |
| HR | 0.0456 | 0.0880 | 0.0745 |
| BR | 0.0408 | 0.0862 | 0.0722 |
| BB | 0.0440 | 0.0928 | 0.0781 |
| FDL | -0.0006 | 0.0043 | 0.0026 |
| SU | -0.0353 | 0.0265 | -0.0114 |
| FU | -0.0190 | 0.0146 | -0.0037 |
| ECU | -0.0220 | 0.0141 | -0.0063 |
| EDL | -0.0258 | 0.0164 | -0.0077 |
| *Crocodylus* |  |  |  |
| Triceps (all) | -0.0355 | -0.0287 | -0.0336 |
| ECR | -0.0104 | -0.0022 | -0.0082 |
| PT | -0.0057 | -0.0035 | -0.0051 |
| AR | -0.0133 | 0.0036 | -0.0078 |
| HR | 0.0377 | 0.0627 | 0.0550 |
| BR | 0.0240 | 0.0370 | 0.0328 |
| BB | 0.0285 | 0.0430 | 0.0381 |
| FDL | -0.0099 | 0.0183 | 0.0061 |
| SU | -0.0073 | 0.0020 | -0.0040 |
| FU | -0.0155 | -0.0104 | -0.0138 |
| ECU | -0.0127 | -0.0012 | -0.0094 |
| EDL | -0.0055 | -0.0032 | -0.0048 |
