# Supplementary material for: Forelimb muscle and joint actions in Archosauria: insights from Crocodylus johnstoni (Pseudosuchia) and Mussaurus patagonicus (Sauropodomorpha)
Source: PeerJ. 2017 Nov 24;5:e3976. doi: 10.7717/peerj.3976 (PMC5703147; doi:10.7717/peerj.3976)
Supplement: Supplemental Information 6 [file peerj-05-3976-s006.docx]

**Table S6**. Results for metacarpo-phalangeal and inter-phalangeal joint moment arms (in metres) of major muscle groups for digit one of *Mussaurus.*

| Moment arms (m) | | | |
| --- | --- | --- | --- |
| Metacarpo-phalangeal | | | |
| Extension (-)/flexion (+) | | | |
| Muscle | Min | Max | Mean |
| FDL | 0.0214 | 0.0339 | 0.0299 |
| FDP | 0.0096 | 0.0272 | 0.0235 |
| FDS | 0.0172 | 0.0279 | 0.0250 |
| EDP | -0.0179 | 0.0030 | -0.0140 |
| EDS | -0.0168 | 0.0027 | -0.0129 |
| Inter-phalangeal | | | |
| FDL | 0.0058 | 0.0340 | 0.0288 |
| EDP | -0.0267 | -0.016 | -0.0232 |
| EDS | -0.0269 | -0.017 | -0.0236 |
