# Supplementary material for: Forelimb muscle and joint actions in Archosauria: insights from Crocodylus johnstoni (Pseudosuchia) and Mussaurus patagonicus (Sauropodomorpha)
Source: PeerJ. 2017 Nov 24;5:e3976. doi: 10.7717/peerj.3976 (PMC5703147; doi:10.7717/peerj.3976)
Supplement: Supplemental Information 7 [file peerj-05-3976-s007.docx]

**Table S7**. Results for elbow and wrist joint moment arms (in metres) of major muscle groups in the reference pose for *Mussaurus* and *Crocodylus.*

|  | Moment arms (m) | | | | | | |  |
| --- | --- | --- | --- | --- | --- | --- | --- | --- |
|  | Elbow | | | Wrist | | | |  |
|  | Extension (-)/flexion (+) | | | Extension (-)/flexion (+) | | | |  |
| *Mussaurus* |  | | |  | | | |  |
| Muscle | Min | Max | Mean | Min | | Max | Mean |  |
| Triceps (all) | -0.0642 | -0.0135 | -0.0498 | – | | – | – |  |
| PT | 0.00082 | 0.0074 | 0.0056 | – | | – | – |  |
| AR | -0.0349 | 0.0333 | -0.0064 | – | | – | – |  |
| HR | 0.0253 | 0.0880 | 0.0685 | – | | – | – |  |
| BR | 0.0215 | 0.0862 | 0.0659 | – | | – | – |  |
| BB | 0.0240 | 0.0928 | 0.0713 | – | | – | – |  |
| FU | -0.0190 | 0.0185 | -0.0006 | – | | – | – |  |
| SU | -0.0353 | 0.0337 | -0.0050 | – | | – | – |  |
| FDL | -0.0020 | 0.0043 | 0.0020 | -0.0224 | | 0.0364 | 0.0226 |  |
| ECU | -0.0220 | 0.0194 | -0.0027 | -0.0370 | | -0.0055 | -0.0297 |  |
| EDL | -0.0258 | 0.0227 | -0.0035 | | -0.0469 | -0.0197 | -0.0404 | |
| ECR | -0.0300 | 0.0284 | -0.0019 | -0.0435 | | -0.0034 | -0.0358 |  |
| APL | – | – | – | -0.0175 | | -0.0028 | -0.0137 |  |
| *Crocodylus* |  |  |  |  | |  |  |  |
| Triceps (all) | -0.0355 | -0.0161 | -0.0322 | – | | – | – |  |
| PT | -0.0057 | -0.0031 | -0.0049 | – | | – | – |  |
| AR | -0.0133 | 0.0077 | -0.0059 | – | | – | – |  |
| HR | 0.0281 | 0.0627 | 0.0520 | – | | – | – |  |
| BR | 0.0194 | 0.0370 | 0.0314 | – | | – | – |  |
| BB | 0.0213 | 0.0430 | 0.0363 | – | | – | – |  |
| FU | -0.0155 | -0.0066 | -0.0131 | – | | – | – |  |
| SU | -0.0073 | 0.0041 | -0.0030 | – | | – | – |  |
| FDL | -0.0099 | 0.0190 | 0.0078 | -0.0034 | | 0.0206 | 0.0130 |  |
| ECU | -0.0127 | 0.0026 | -0.0080 | -0.0127 | | 0.0024 | -0.0078 |  |
| EDL | -0.0055 | -0.0031 | -0.0046 | -0.0465 | | -0.0276 | -0.0406 |  |
| ECR | -0.0104 | 0.0007 | -0.0072 | -0.0098 | | 0.0051 | -0.0053 |  |
| APL | – | – | – | -0.0209 | | 0.0110 | -0.0154 |  |
