# Supplementary material for: Forelimb muscle and joint actions in Archosauria: insights from Crocodylus johnstoni (Pseudosuchia) and Mussaurus patagonicus (Sauropodomorpha)
Source: PeerJ. 2017 Nov 24;5:e3976. doi: 10.7717/peerj.3976 (PMC5703147; doi:10.7717/peerj.3976)
Supplement: Supplemental Information 8 [file peerj-05-3976-s008.docx]

**Table S8**. Muscle actions for *Crocodylus* and *Mussaurus* in the reference pose.

|  | Glenoid | | | | | | Elbow | |
| --- | --- | --- | --- | --- | --- | --- | --- | --- |
|  | Pronation/supination | | Extension/flexion | | Abduction/adduction | | Extension/flexion | |
| Muscle | *Crocodylus* | *Mussaurus* | *Crocodylus* | *Mussaurus* | *Crocodylus* | *Mussaurus* | *Crocodylus* | *Mussaurus* |
| DS | supination | supination | flexion | flexion | abduction | abduction | — | — |
| DC | supination | supination | flexion | flexion | abduction | abduction | — | — |
| TM | supination | supination | extension | extension | abduction | abduction | — | — |
| SBS | pronation | pronation | extension | extension | **abduction** | **adduction** | — | — |
| SHP | **mixed** | **pronation** | **extension** | **mixed** | abduction | abduction | — | — |
| SCI | supination | supination | flexion | flexion | adduction | adduction | — | — |
| SCB | supination | supination | flexion | flexion | adduction | adduction | — | — |
| SCL | supination | supination | flexion | flexion | adduction | adduction | — | — |
| CBV | **pronation** | **mixed** | **extension** | **flexion** | adduction | adduction | — | — |
| CBD | supination | supination | flexion | flexion | **adduction** | **mixed** | — | — |
| TBS | **pronation** | **mixed** | **extension** | **flexion** | abduction | abduction | extension | extension |
| TBC | mixed | mixed | **extension** | **mixed** | abduction | abduction | extension | extension |
| TBM4 | — | — | — | — | — | — | extension | extension |
| TBM1 | — | — | — | — | — | — | extension | extension |
| TBM3 | — | — | — | — | — | — | extension | extension |
| TBL | — | — | — | — | — | — | extension | extension |
| TBM2 | — | — | — | — | — | — | extension | extension |
| BB | supination | supination | mixed | mixed | **adduction** | **mixed** | flexion | flexion |
| HR | — | — | **—** | **—** | — | — | flexion | flexion |
| BR | — | — | — | — | — | — | flexion | flexion |
| SU | — | — | — | — | — | — | **extension** | **mixed** |
| FU | — | — | — | — | — | — | **extension** | **mixed** |
| AR | — | — | — | — | — | — | **extension** | **mixed** |
| PT | — | — | — | — | — | — | **extension** | **flexion** |
| FDL | — | — | — | — | — | — | **mixed** | **flexion** |
| EDL | — | — | — | — | — | — | **extension** | **mixed** |
| ECR | — | — | — | — | — | — | **extension** | **mixed** |
| ECU | — | — | — | — | — | — | **extension** | **mixed** |

Bold font highlights a difference between the two taxa. ‘—’ indicates that the muscle was inferred not to act around that axis in the model.
