# Supplementary material for: Forelimb muscle and joint actions in Archosauria: insights from Crocodylus johnstoni (Pseudosuchia) and Mussaurus patagonicus (Sauropodomorpha)
Source: PeerJ. 2017 Nov 24;5:e3976. doi: 10.7717/peerj.3976 (PMC5703147; doi:10.7717/peerj.3976)
Supplement: Supplemental Information 9 [file peerj-05-3976-s009.docx]

**Table S9**. Results for shoulder joint moment arms (in metres) of major muscle groups in the reference pose for *Mussaurus* and *Crocodylus.*

| Moment arms (m) | | | | | | | | | |
| --- | --- | --- | --- | --- | --- | --- | --- | --- | --- |
|  | Pronation (-)/supination (+) | | | Abduction (-)/adduction (+) | | | Extension (-)/flexion (+) | | |
| *Mussaurus* |  | | |  | | |  | | |
| Muscle | Min | Max | Mean | Min | Max | Mean | Min | Max | Mean |
| DC | 0.0570 | 0.0757 | 0.0696 | -0.0768 | -0.0550 | -0.0671 | 0.0097 | 0.0230 | 0.0189 |
| DS | 0.0551 | 0.0820 | 0.0716 | -0.0175 | -0.0078 | -0.0130 | 0.0023 | 0.0098 | 0.0072 |
| TM | 0.0378 | 0.0644 | 0.0539 | -0.0584 | -0.0430 | -0.0520 | -0.0231 | 0.0033 | -0.0116 |
| SHP | -0.0031 | -0.0028 | -0.0030 | -0.0305 | -0.0268 | -0.0294 | -0.0054 | 0.0038 | -0.0009 |
| SBS | -0.0711 | -0.0619 | -0.0685 | 0.0143 | 0.0184 | 0.0169 | -0.0181 | 0.0038 | -0.0087 |
| SC (all) | 0.0426 | 0.0651 | 0.0579 | 0.0137 | 0.0187 | 0.0154 | -0.0411 | 0.0576 | 0.0274 |
| CBD | 0.0793 | 0.0983 | 0.0926 | -0.0135 | 0.0042 | -0.0047 | 0.0185 | 0.0386 | 0.0300 |
| CBV | -0.0739 | 0.0411 | -0.0251 | 0.0348 | 0.0454 | 0.0422 | 0.0519 | 0.0690 | 0.0634 |
| TBS | -0.0197 | 0.0070 | -0.0069 | -0.1662 | -0.1331 | -0.1514 | -0.0109 | 0.0368 | 0.0145 |
| TBC | -0.0023 | 0.0038 | 0.0006 | -0.0655 | -0.0398 | -0.0532 | -0.0343 | 0.0241 | -0.0055 |
| BB | 0.0180 | 0.0582 | 0.0413 | -0.0026 | 0.0108 | 0.0022 | -0.0531 | 0.0639 | 0.0245 |
| *Crocodylus* |  |  |  |  |  |  |  |  |  |
| DC | 0.0233 | 0.0357 | 0.0285 | -0.0224 | -0.0056 | -0.0144 | 0.0209 | 0.0233 | 0.0225 |
| DS | 0.0258 | 0.0329 | 0.0308 | -0.0188 | -0.0144 | -0.0177 | 0.0037 | 0.0092 | 0.0072 |
| TM | -0.0059 | 0.0180 | 0.0065 | -0.0328 | -0.0262 | -0.0311 | -0.0098 | -0.0068 | -0.0091 |
| SHP | -0.0111 | 0.0098 | -0.0007 | -0.0273 | -0.0216 | -0.0259 | -0.0077 | -0.0056 | -0.0071 |
| SBS | -0.0320 | -0.0285 | -0.0311 | -0.0131 | -0.0041 | -0.0094 | -0.0218 | -0.0103 | -0.0186 |
| SC (all) | 0.0126 | 0.0261 | 0.0206 | 0.0108 | 0.0248 | 0.0184 | 0.0211 | 0.0523 | 0.0382 |
| CBD | 0.0099 | 0.0162 | 0.0132 | 0.0104 | 0.0166 | 0.0142 | 0.0136 | 0.0173 | 0.0163 |
| CBV | -0.0076 | -0.0037 | -0.0059 | 0.0229 | 0.0329 | 0.0292 | -0.0180 | 0.0052 | -0.0067 |
| TBS | -0.0153 | -0.0018 | -0.0091 | -0.0585 | -0.0335 | -0.0476 | -0.0201 | -0.0002 | -0.0110 |
| TBC | -0.0071 | 0.0315 | 0.0139 | -0.0417 | -0.0357 | -0.0400 | -0.0480 | -0.0017 | -0.0367 |
| BB | 0.0152 | 0.0239 | 0.0177 | 0.0217 | 0.0319 | 0.0280 | -0.0087 | 0.0264 | 0.0090 |
