# Supplementary material for: Forelimb muscle and joint actions in Archosauria: insights from Crocodylus johnstoni (Pseudosuchia) and Mussaurus patagonicus (Sauropodomorpha)
Source: PeerJ. 2017 Nov 24;5:e3976. doi: 10.7717/peerj.3976 (PMC5703147; doi:10.7717/peerj.3976)
Supplement: Supplemental Information 10 [file peerj-05-3976-s010.docx]

**Table S10**. Results of the sensitivity analysis for elbow joint moment arms (in metres) of the major extensor muscle group in the resting pose in *Mussaurus.*

| Moment arms (m) | | | | | | | | | | | |
| --- | --- | --- | --- | --- | --- | --- | --- | --- | --- | --- | --- |
| Elbow (-25%) | | | Elbow (not altered) | | | | | | Elbow (+25%) | | |
| Extension (-)/flexion (+) | | | | | | | | | | | |
| Muscle | Min | Max | | Mean | Min | Max | Mean | Min | | Max | Mean |
| Triceps  (all) | -0.0564 | -0.0281 | | -0.0470 | -0.0651 | -0.0317 | -0.0556 | -0.0720 | | -0.0349 | -0.0632 |
