# Supplementary material for: Forelimb muscle and joint actions in Archosauria: insights from Crocodylus johnstoni (Pseudosuchia) and Mussaurus patagonicus (Sauropodomorpha)
Source: PeerJ. 2017 Nov 24;5:e3976. doi: 10.7717/peerj.3976 (PMC5703147; doi:10.7717/peerj.3976)
Supplement: Supplemental Information 11 — For additional muscle abbreviations and details see Table 1 and Table S1’s caption. [file peerj-05-3976-s011.docx]

**Table S11**. Wrapping surfaces used for the musculoskeletal model of *Mussaurus patagonicus* in the sensitivity analysis. For additional muscle abbreviations and details see Table 1 and Table S1’s caption.

| Minus 25% |  |  |  |  |  |  |  |  |  |  |
| --- | --- | --- | --- | --- | --- | --- | --- | --- | --- | --- |
| Muscle involved | Location | Shape | r(x) | r(y) | r(z) | t(x) | t(y) | t(z) | Radius | Height |
| TBM3 | Distal humerus | Cylinder | -9.07 | 1.88 | -86.43 | 0.4009 | -0.0014 | 0.0044 | 0.0234 | 0.195 |
| TBS | Distal humerus | Cylinder | -12.14 | 2.8 | -88.58 | 0.3990 | -0.0060 | -0.0087 | 0.0234 | 0.195 |
| TBM4, TBL | Distal humerus | Cylinder | 168.57 | -2.62 | -109.1 | 0.3998 | -0.0034 | -0.0096 | 0.0234 | 0.0975 |
| TBM2 | Distal humerus | Cylinder | 165.23 | -0.54 | 70.06 | 0.3991 | -0.0058 | -0.0090 | 0.0234 | 0.195 |
| TBM1, TBC | Distal humerus | Cylinder | -13.22 | 3.87 | 73.46 | 0.4005 | -0.0056 | -0.0042 | 0.0234 | 0.195 |
| Plus 25% |  |  |  |  |  |  |  |  |  |  |
| Muscle involved | Location | Shape | r(x) | r(y) | r(z) | t(x) | t(y) | t(z) | Radius | Height |
| TBM3 | Distal humerus | Cylinder | -9.07 | 1.88 | -86.43 | 0.4009 | 0.0014 | 0.0044 | 0.039 | 0.195 |
| TBS | Distal humerus | Cylinder | -12.14 | 2.8 | -88.58 | 0.3990 | -0.0060 | -0.0087 | 0.039 | 0.195 |
| TBM4, TBL | Distal humerus | Cylinder | 168.57 | -2.62 | -109.1 | 0.3998 | -0.0034 | -0.0096 | 0.039 | 0.0975 |
| TBM2 | Distal humerus | Cylinder | 165.23 | -0.54 | 70.06 | 0.3991 | -0.0058 | -0.0090 | 0.039 | 0.195 |
| TBM1, TBC | Distal humerus | Cylinder | -13.22 | 3.87 | 73.46 | 0.4005 | -0.0056 | -0.0042 | 0.039 | 0.195 |
