# Supplementary material for: Forelimb muscle and joint actions in Archosauria: insights from Crocodylus johnstoni (Pseudosuchia) and Mussaurus patagonicus (Sauropodomorpha)
Source: PeerJ. 2017 Nov 24;5:e3976. doi: 10.7717/peerj.3976 (PMC5703147; doi:10.7717/peerj.3976)
Supplement: Supplemental Information 12 [file peerj-05-3976-s012.docx]

**Table S12**. Results for elbow joint moment arms (in metres) of major muscle groups in the resting pose in *Mussaurus* in a pronated position (by 30°) for long axis rotation.

| Moment arms (m) | | | |
| --- | --- | --- | --- |
| Elbow | | | |
| Pronation (-)/supination (+) | | | |
| Muscle | Min | Max | Mean |
| Triceps (all) | 0.0192 | 0.0573 | 0.0369 |
| BB | 0.0091 | 0.0181 | 0.0140 |
| HR | 0.0057 | 0.0169 | 0.0116 |
| BR | 0.0181 | 0.0219 | 0.0205 |
| FU | -0.0244 | -0.0135 | -0.0196 |
| ECR | 0.0024 | 0.0041 | 0.0033 |
| AR | 0.0199 | 0.0228 | 0.0219 |
| SU | 0.0168 | 0.0225 | 0.0203 |
| PT | 0.0103 | 0.0297 | 0.0211 |
| FDL | 0.0001 | 0.0111 | 0.0057 |
| EDL | 0.0032 | 0.0034 | 0.0033 |
| ECU | 0.0007 | 0.0030 | 0.0019 |
