# Supplementary figures and images for: Forelimb muscle and joint actions in Archosauria: insights from Crocodylus johnstoni (Pseudosuchia) and Mussaurus patagonicus (Sauropodomorpha)
Source: PeerJ. 2017 Nov 24;5:e3976. doi: 10.7717/peerj.3976 (PMC5703147; doi:10.7717/peerj.3976)

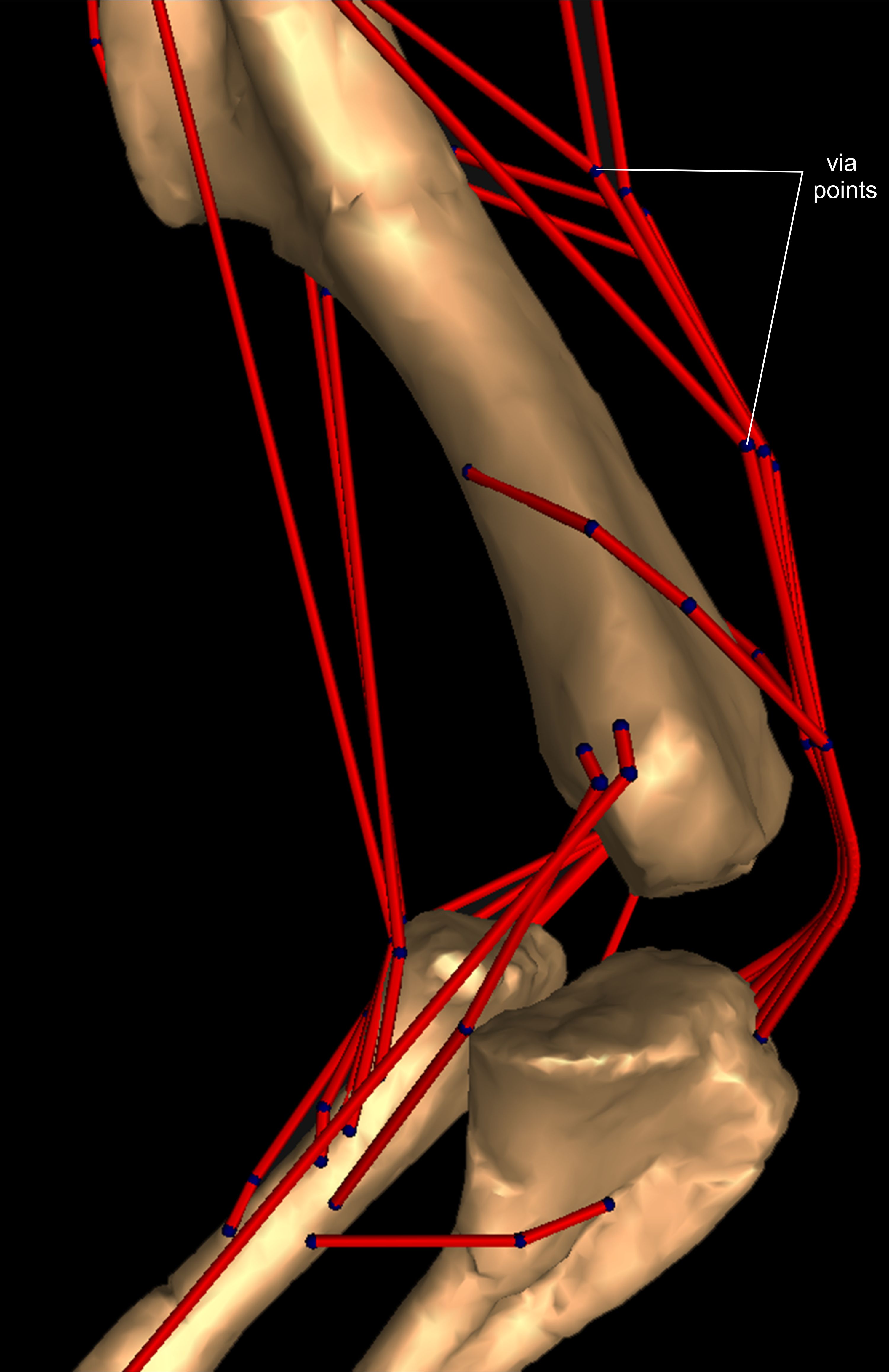

Supplement: Supplemental Information 13 [file peerj-05-3976-s013.jpg]

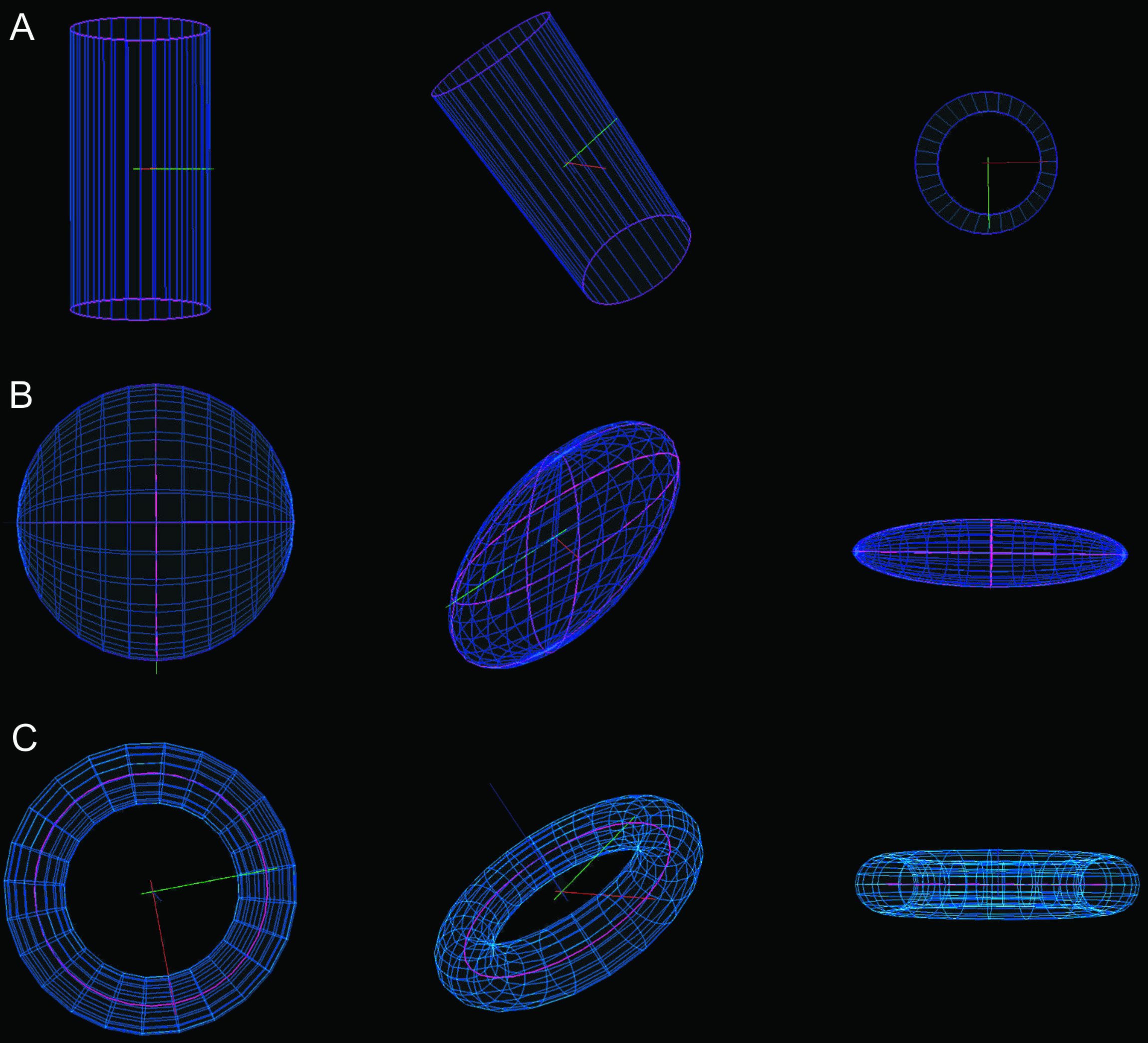

Supplement: Supplemental Information 14 [file peerj-05-3976-s014.jpg]

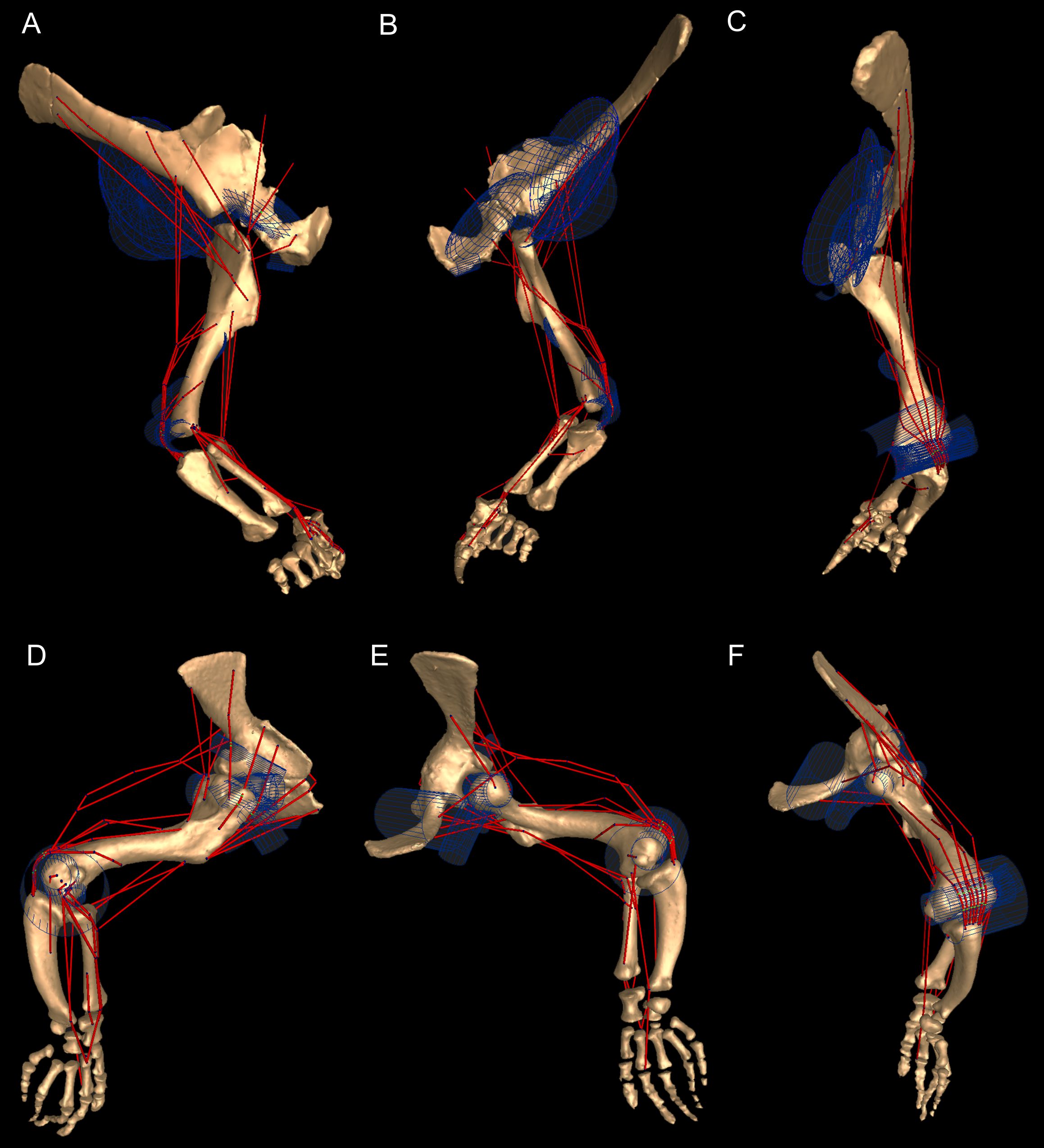

Supplement: Supplemental Information 15 — Three-dimensional musculoskeletal models of the right forelimbs of Mussaurus patagonicus (A–C) and Crocodylus johnstoni (D–F) in the resting pose, showing wrapping objects used in this study in lateral (A, D), medial (B, E) and caudomedial (C, F) views. Scale bar: 10 cm. Compare with Fig. 3. [file peerj-05-3976-s015.jpg]

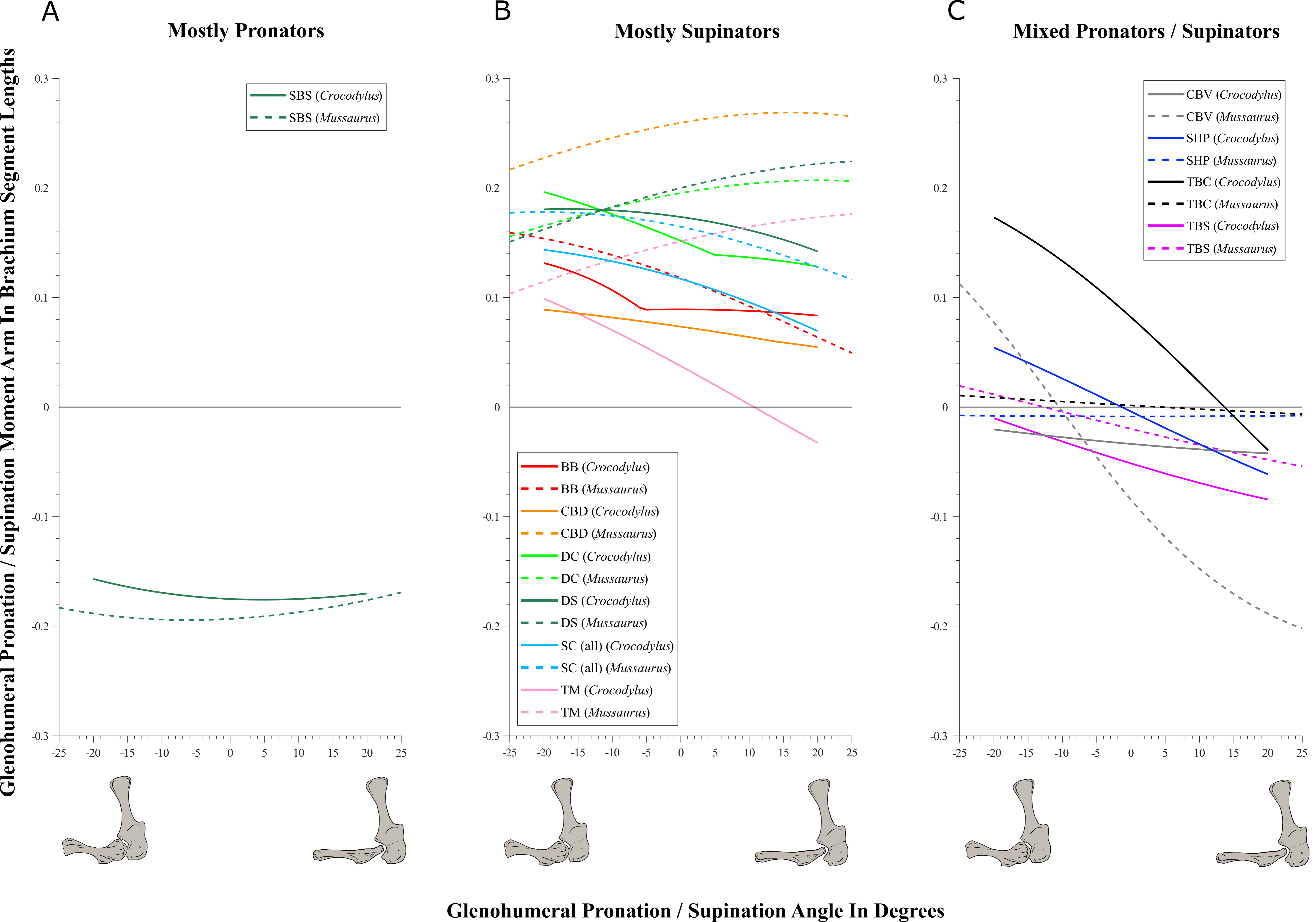

Supplement: Supplemental Information 16 — (A) mostly pronator; (B) mostly supinators; (C) mixed pronators/supinators. Negative moment arms and glenohumeral angles correspond to pronation, while positive values correspond to supination. For muscle abbreviations see Table 1. [file peerj-05-3976-s016.pdf]

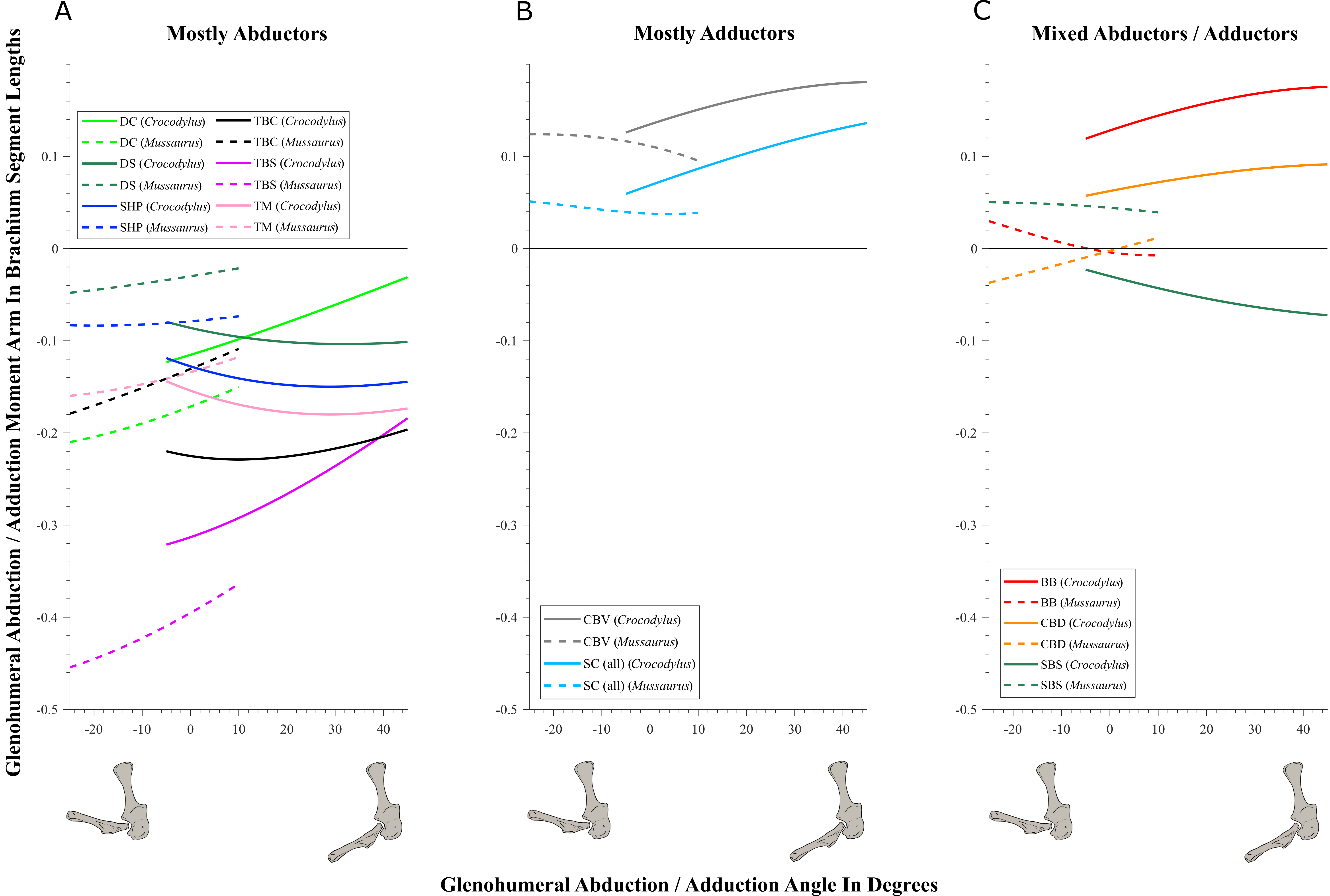

Supplement: Supplemental Information 18 — (A) mostly abductors; (B) mostly adductors; (C) mixed abductors/adductors. Negative moment arms and glenohumeral angles correspond to abduction, while positive values correspond to adduction. For muscle abbreviations see Table 1. [file peerj-05-3976-s018.pdf]

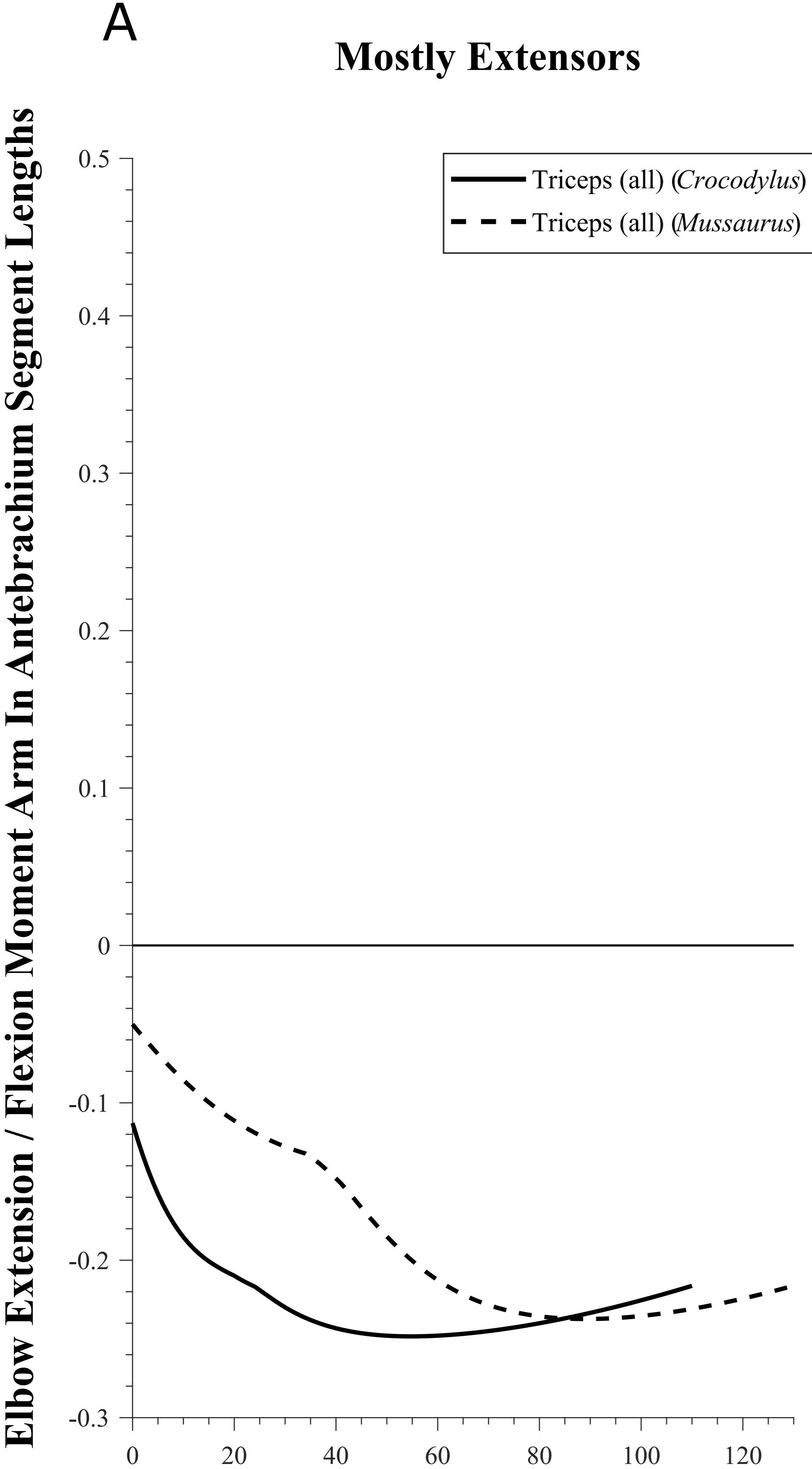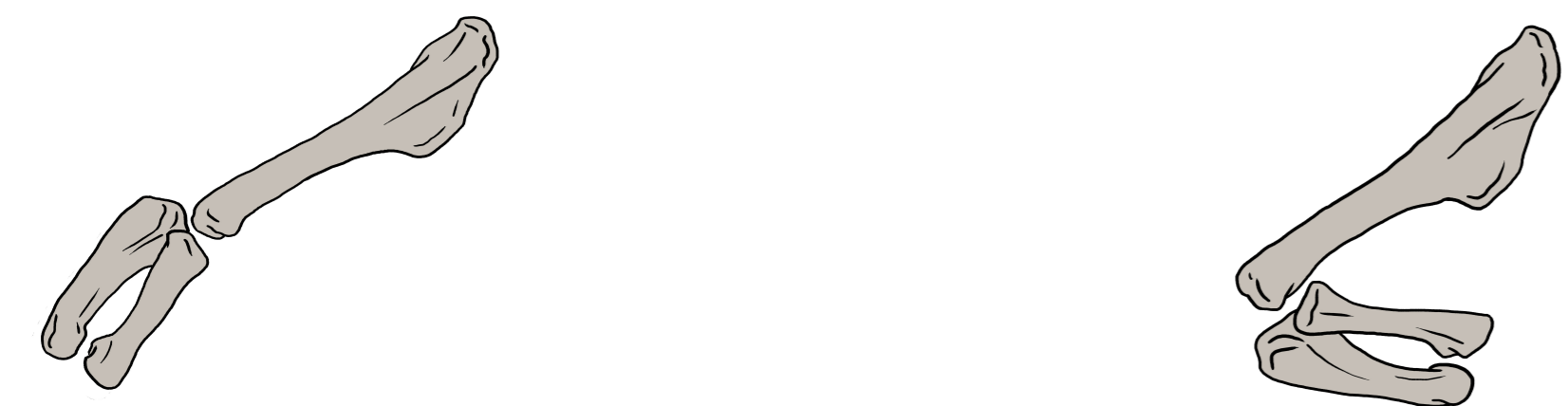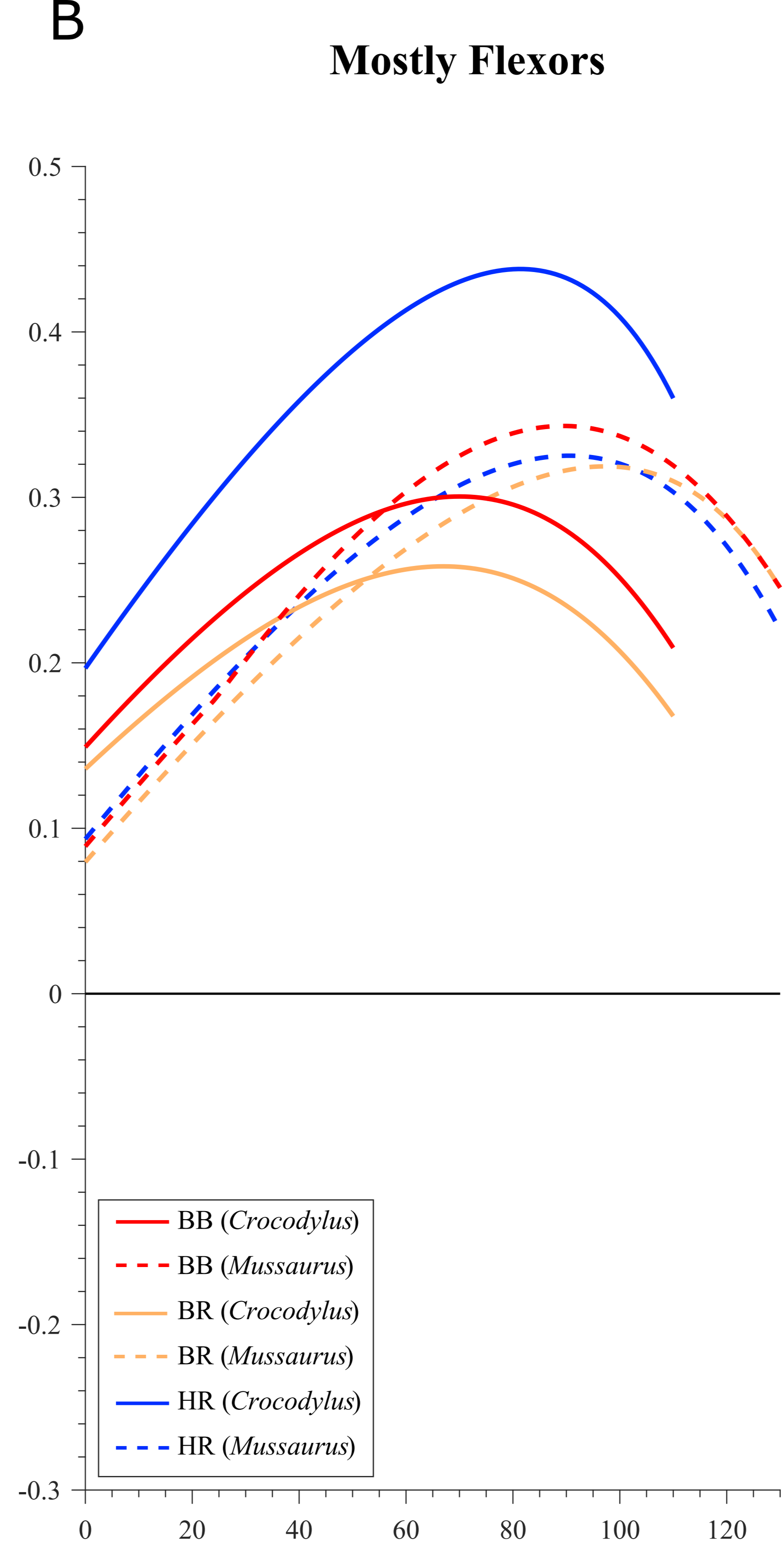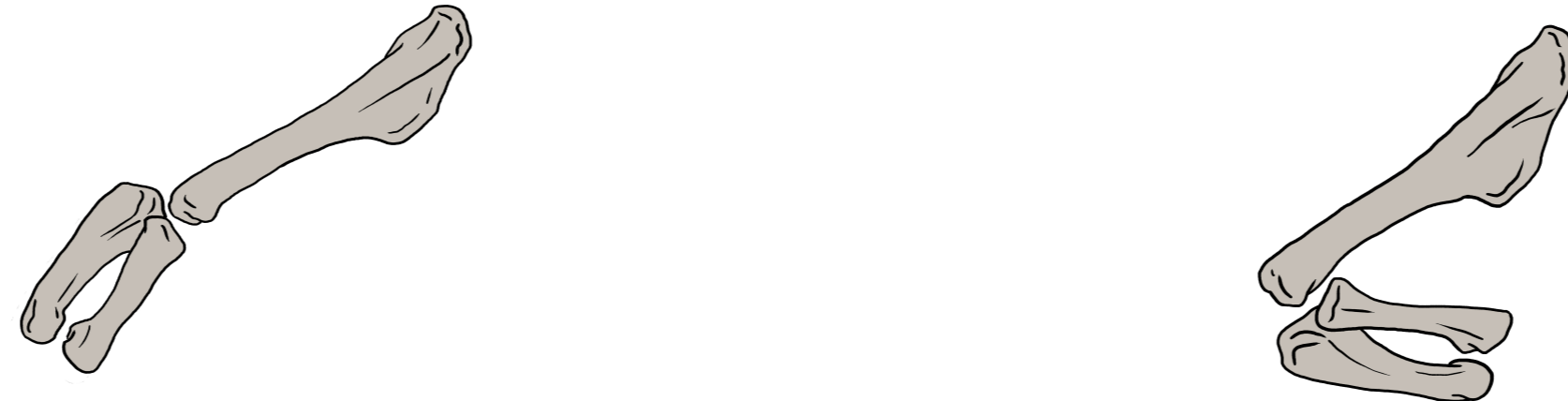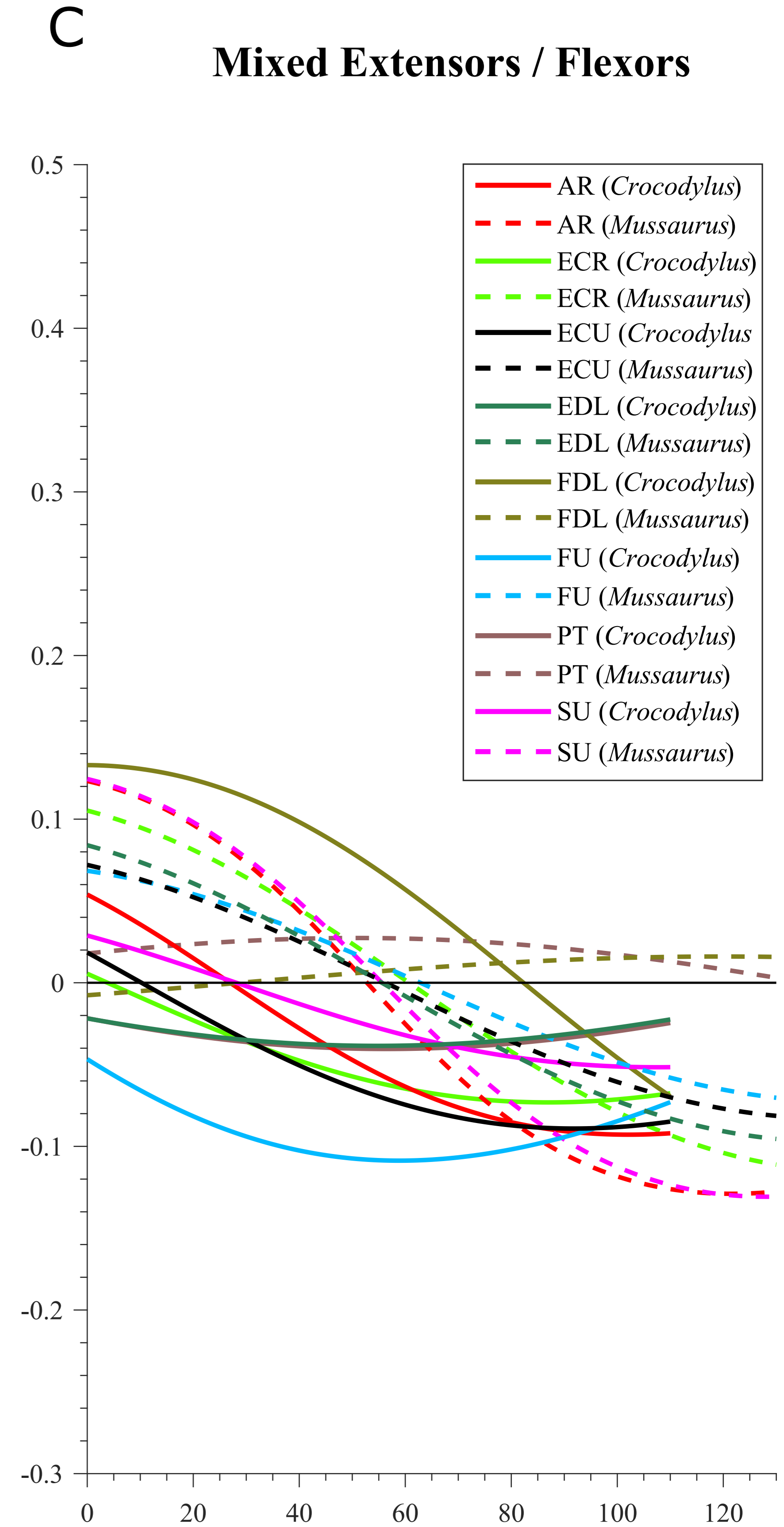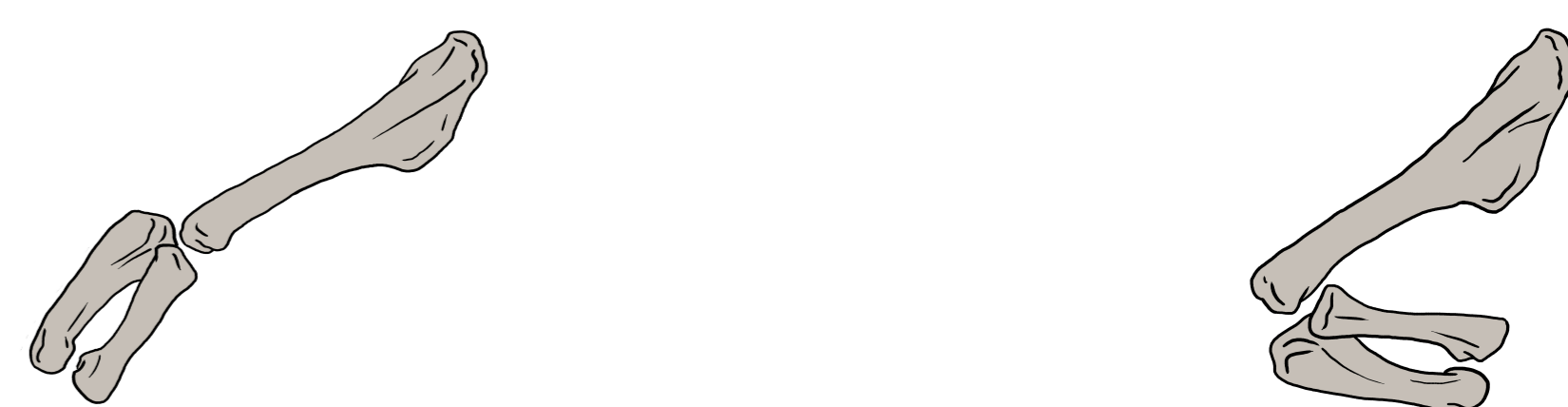

Elbow Extension / Flexion Joint Angle In Degrees

Supplement: Supplemental Information 19 — (A) extensors; (B) flexors; (C) mixed extensors/flexors. Negative moment arms correspond to extension, while positive values correspond to flexion. Zero elbow angle corresponds to full extension, while larger angles correspond to flexion. For muscle abbreviations see Table 1. [file peerj-05-3976-s019.pdf]

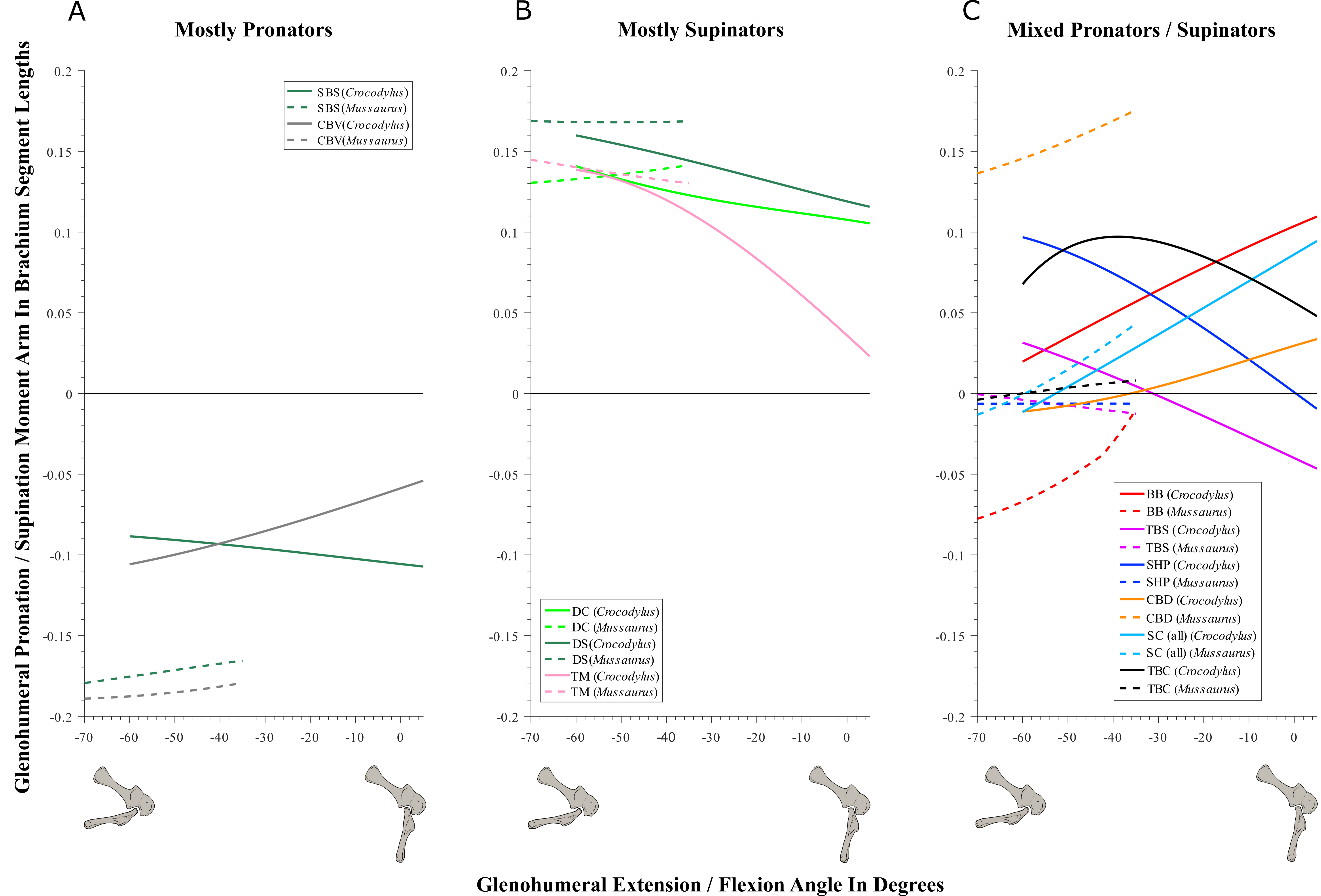

Supplement: Supplemental Information 20 — (A) mostly pronators; (B) mostly supinators; (C) mixed pronators/supinators. Negative moment arms and glenohumeral angles correspond to pronation, while positive values correspond to supination. For muscle abbreviations see Table 1. [file peerj-05-3976-s020.pdf]

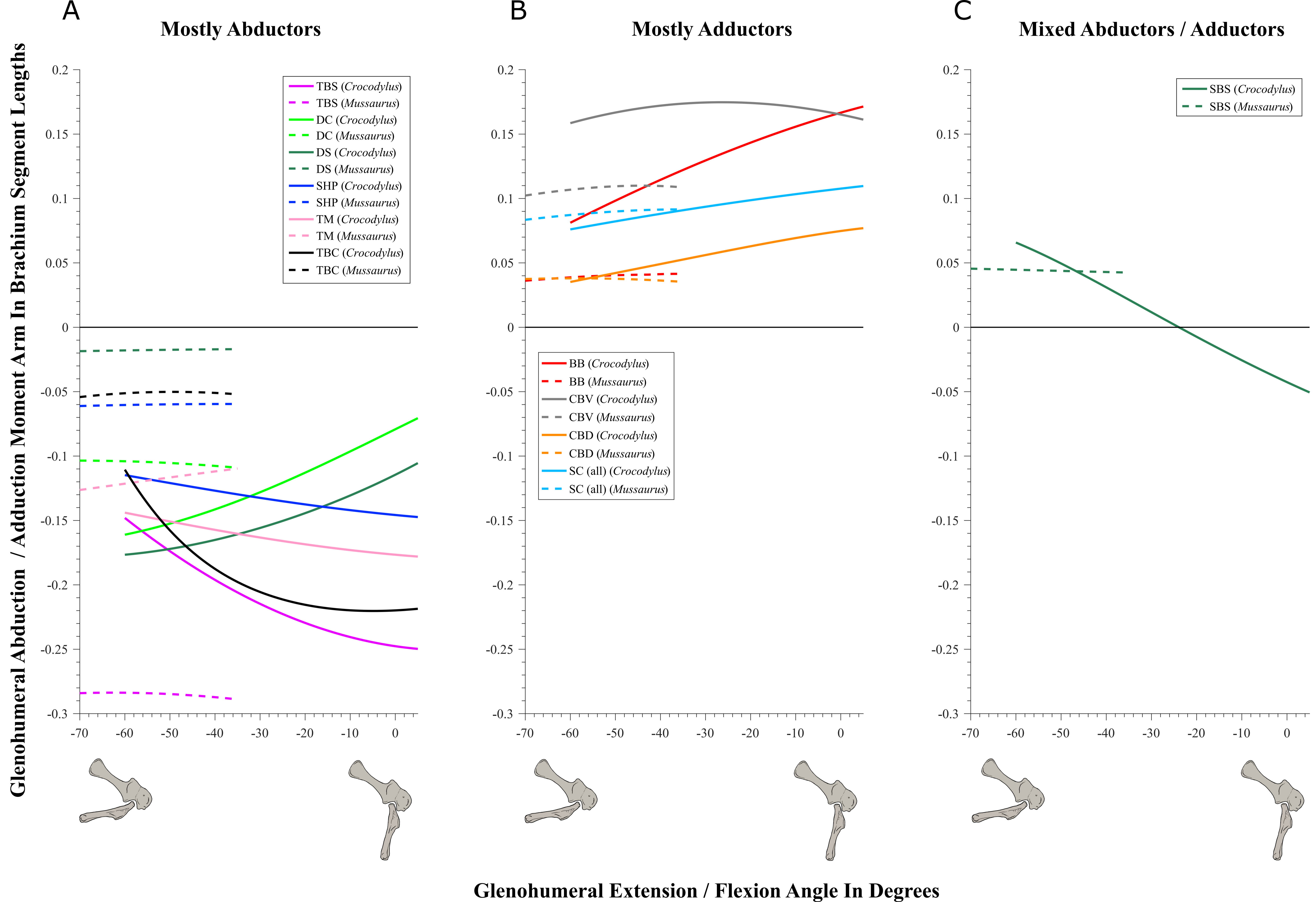

Supplement: Supplemental Information 21 — (A) mostly abductors; (B) mostly adductors; (C) mixed abductors/adductors. Negative moment arms and glenohumeral angles correspond to abduction, while positive values correspond to adduction. For muscle abbreviations see Table 1. [file peerj-05-3976-s021.pdf]

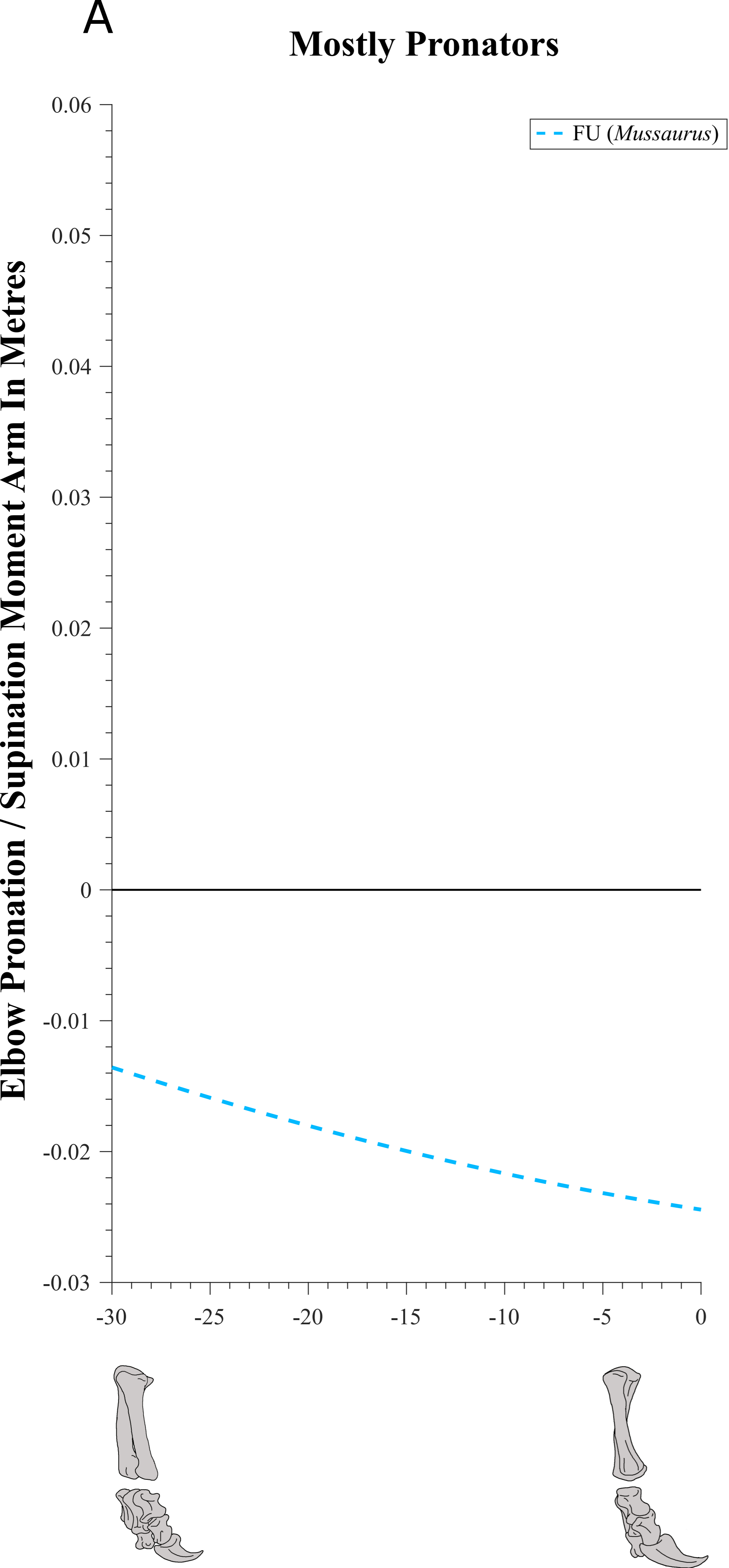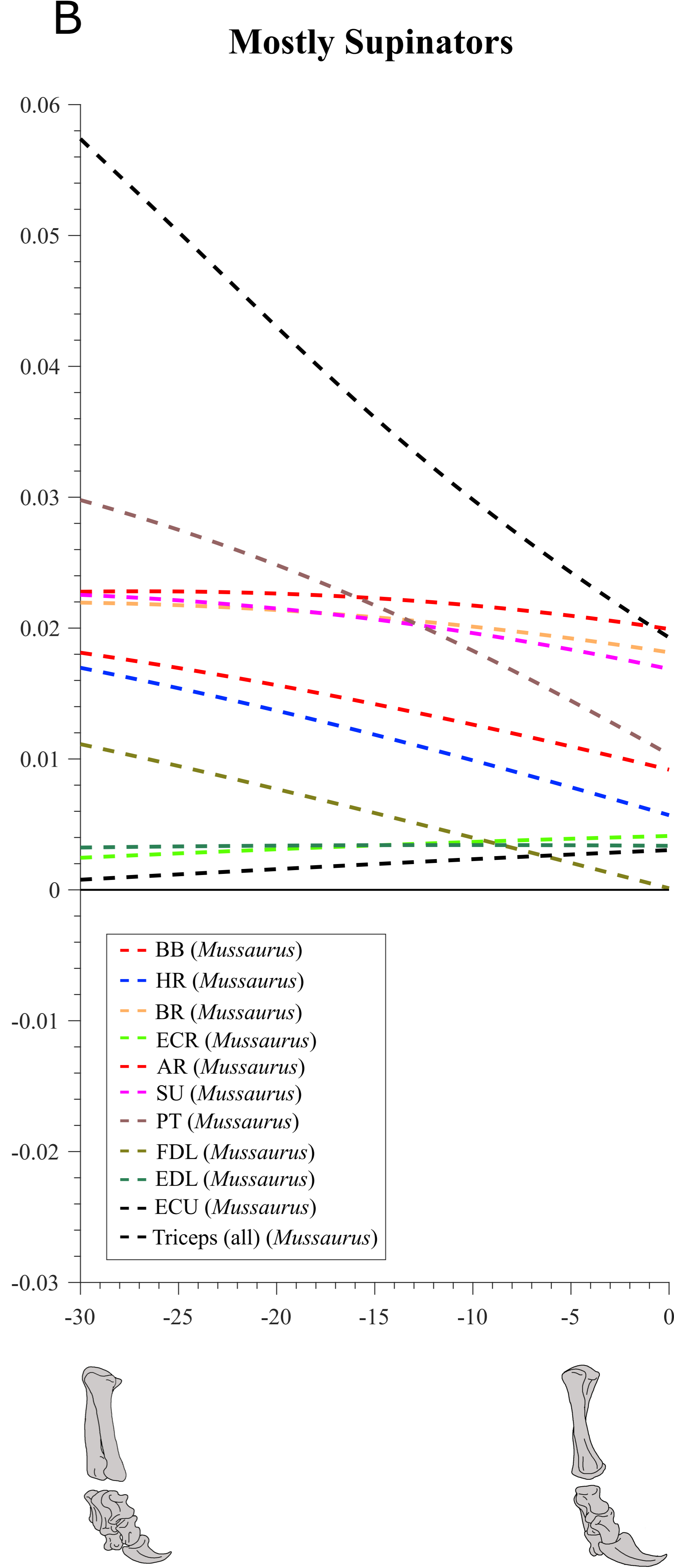

Supplement: Supplemental Information 22 — (A) mostly pronators; (B) mostly supinators; (C) mixed pronators/supinators. Negative moment arms and joint angles correspond to pronation, whilst positive values correspond to supination. Zero elbow angle corresponds to a neutral position in between pronation/supination. For muscle abbreviations see Table 1. [file peerj-05-3976-s022.pdf]
