# Supplementary material for: Forelimb muscle and joint actions in Archosauria: insights from Crocodylus johnstoni (Pseudosuchia) and Mussaurus patagonicus (Sauropodomorpha)
Source: PeerJ. 2017 Nov 24;5:e3976. doi: 10.7717/peerj.3976 (PMC5703147; doi:10.7717/peerj.3976)
Supplement: Supplemental Information 17 — (A) mostly extensors; (B) mostly flexors; (C) mixed extensors/flexors. Negative moment arms and glenohumeral angles correspond to extension, while positive values correspond to flexion. For muscle abbreviations see Table 1. [file peerj-05-3976-s017.pdf]

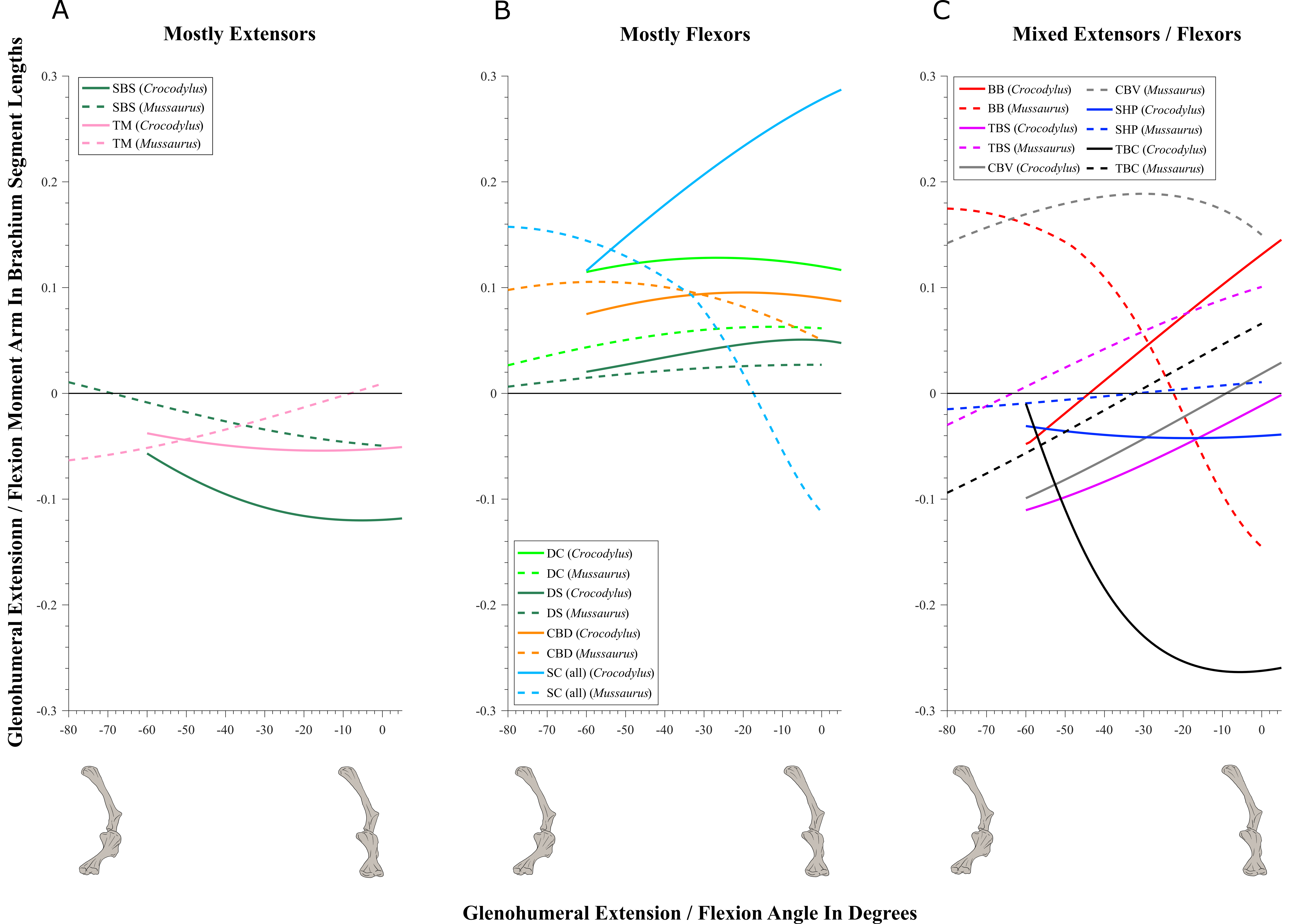

A
Mostly Extensors
B
Mostly Flexors
C
Mixed Extensors / Flexors
Glenohumeral Extensionn / Flexion Moment Arm In Brachium Segment Lengths
SBS (Crocodylus)
SBS (Mussaurus)
TM (Crocodylus)
TM (Mussaurus)
DC (Crocodylus)
DC (Mussaurus)
DS (Crocodylus)
DS (Mussaurus)
CBD (Crocodylus)
CBD (Mussaurus)
SC (all) (Crocodylus)
SC (all) (Mussaurus)
BB (Crocodylus)
BB (Mussaurus)
TBS (Crocodylus)
TBS (Mussaurus)
CBV (Crocodylus)
CBV (Mussaurus)
SHP (Crocodylus)
SHP (Mussaurus)
TBC (Crocodylus)
TBC (Mussaurus)
0.3
0.2
0.1
0
-0.1
-0.2
-0.3
-80
-70
-60
-50
-40
-30
-20
-10
0
Glenohumeral Extension / Flexion Angle In Degrees
