# Supplementary material for: Forelimb muscle and joint actions in Archosauria: insights from Crocodylus johnstoni (Pseudosuchia) and Mussaurus patagonicus (Sauropodomorpha)
Source: PeerJ. 2017 Nov 24;5:e3976. doi: 10.7717/peerj.3976 (PMC5703147; doi:10.7717/peerj.3976)
Supplement: Supplemental Information 23 [file peerj-05-3976-s023.docx]

**Supplementary Text**

**Forelimb muscle and joint actions in Archosauria: insights from *Crocodylus johnstoni* (Pseudosuchia) and *Mussaurus patagonicus* (Sauropodomorpha)**

Alejandro Otero, Vivian Allen, Diego Pol, John R. Hutchinson

**Moment arm differences between the reference and the resting pose**

We expected to find some differences in muscle actions when comparing the reference (Figs. S4–S7; Table S8) and the resting (Figs. 5–8; Tables 3) forelimb poses. Indeed, we found some differences, as follows; additionally, magnitudes of muscle moment arms changed in many cases but we do not detail these here.

Long axis rotation did not exhibit substantial differences in muscle actions for the glenohumeral joint’s reference vs. resting poses in *Mussaurus* (Figs. 5, S4), except for SHP and CBV, which had mixed actions in the reference pose but became solely pronators in the resting pose, and the BB, which was a supinator in the reference pose, but displayed a mixed supinator/pronator action in the resting pose. In *Crocodylus*, SHP had a mixed action in the reference pose (Fig. S4) but was solely a supinator in the resting pose (Fig. 5). Similarly, TBS displayed a fully pronator action in the reference pose, but shifted to supination near 10° of glenohumeral supination when using the resting pose model.

The muscles of *Crocodylus* showed no substantial changes for flexion/extension of the glenohumeral joint between the reference (Fig. S5) and resting poses (Fig. 6), except for BB, which was a mixed muscle in the reference pose and fully a flexor in the resting pose (Fig. 6). *Mussaurus*, on the other hand, in addition to the same changes for BB as in *Crocodylus*, exhibited drastic differences in muscle actions, particularly for the SHP and triceps group. The TBC had a mixed action in the reference pose, but was only an extensor in the resting pose. The SHP displayed a mixed action in the reference pose, but shifted to a glenohumeral extensor action when the resting pose was adopted. Conversely, TBS consistently acted as a flexor in the reference pose in *Mussaurus* but was converted to a mixed action muscle in the resting pose.

Muscle abductor/adductor actions around the glenohumeral joint also differed between the reference vs. resting poses (Figs. S6, 7, respectively) for both taxa. In *Crocodylus*, SBS was an abductor in the reference pose but became an adductor in the resting pose. In *Mussaurus*, BB had a mixed action in the reference pose, but was a glenohumeral adductor in the resting pose (Figs. S6 and 7, respectively).

The elbow joint showed no pronounced changes in muscle actions for the moderately different reference and resting poses in either taxon.
